# Treatment-Free Remission in Chronic Myeloid Leukemia

**DOI:** 10.3390/jcm13092567

**Published:** 2024-04-27

**Authors:** Garrett Bourne, Ravi Bhatia, Omer Jamy

**Affiliations:** Department of Medicine, University of Alabama at Birmingham, Birmingham, AL 35294, USA

**Keywords:** chronic myeloid leukemia, tyrosine kinase inhibitors, treatment-free remission

## Abstract

With the discovery of tyrosine kinase inhibitors (TKIs), overall survival in patients with chronic myeloid leukemia (CML) now approaches that of the general population. While these TKIs have proven to be lifesaving, remaining on them lifelong creates both physical and financial burdens for patients. Recently, multiple trials have begun looking into the efficacy of trialing patients off these TKIs to see if they can sustain treatment-free remission (TFR). TFR eligibility is currently limited to a small population of patients with both robust and sustained responses to TKIs. Currently, for those who attempt a trial of TFR, the average success rates are promising, with anywhere from 38 to 54% of patients experiencing sustained TFR. For those who fail to maintain sustained TFR, safety results to date are reassuring, with almost all patients successfully responding to the re-initiation of TKIs, with death and disease progression being very rare complications. Moving forward, research is being conducted to more accurately risk stratify patients at diagnosis and pair them with optimized upfront treatment regimens aimed at increasing candidacy for the trial of TFR.

## 1. Introduction

Chronic myeloid leukemia (CML) is a myeloproliferative neoplasm that is heralded by many as one of the great successes of modern medicine [1]. The disease arises from a specific reciprocal chromosomal translocation, (t(9;22) (q34;q11)), now known as the Philadelphia chromosome (Ph) [2]. Upon chromosomal translocation, the *BCR* gene on chromosome 22 and the *ABL1* gene on chromosome 9 are brought within proximity, facilitating the creation of the fusion *BCR-ABL1* oncogene. This oncogene creates the constitutively active tyrosine kinase protein, *BCR-ABL1*, that results in hematopoietic stem cell transformation and uncontrolled hematopoietic cell proliferation [2]. In the very early years of CML management, the average 8-year survival rate was estimated to have been <20% [3]. However, in 2001, the FDA approved imatinib, a novel therapeutic that specifically inhibited the *BCR-ABL1* tyrosine kinase [4]. The long-term follow-up of patients with CML treated with imatinib revealed a 10-year overall survival rate of 83% [3,5,6].

While survival rates increased, many patients taking imatinib experienced fatigue, fluid retention, rashes, gastrointestinal (GI) toxicities, muscle aches and cytopenias [7]. With a hope of minimizing side effects, overcoming drug resistance and increasing survival rates even further, second- and now third-generation tyrosine kinase inhibitors (TKIs) have become commercially available. Although many of these advanced-generation TKIs have been shown to provide deeper remissions over a shorter time period, there has been no proven overall survival (OS) benefit relative to imatinib [8]. Furthermore, second- and third-generation TKIs have their own unique toxicity profiles and may have increased risk of serious cardiovascular or pulmonary toxicities compared to imatinib. Rather, it has been observed that side effect tolerability between TKIs varies from patient to patient [8]. Another limitation of TKIs is that they fail to eliminate the primitive, quiescent leukemia stem cells responsible for propagating CML. Therefore, CML patients remain at risk for leukemia relapse after TKI discontinuation and may require continued life-long TKI treatment to prevent recurrence. Furthermore, it is an unfortunate but well-known fact that TKIs are associated with financial toxicity [9]. Average annual costs have been reported to be upward of USD 200,000 per patient, with some estimates quoting an approximate 5 billion total USD spent annually in the US healthcare system on these drugs [10,11]. Despite insurance and various payment programs, it is not uncommon for patients to face financial burden during their CML treatment. Table 1 summarizes some of the most common side effects and approximated costs of currently available TKIs.

Novel TKIs changed the landscape of CML so profoundly that many considered them a virtual cure [1]. However, if patients are required to take a medicine indefinitely that comes with its own burden of both physical and financial toxicity, is the disease actually cured? This question has led to attempts at TKI discontinuation that entail stopping all CML directed therapy and monitoring for recurrence in patients who have shown sustained deep molecular response (DMR) with TKIs. If pathways are discovered that consistently progress patients into sustained treatment-free remission (TFR), only then is a cure for CML truly being developed. In this review, we discuss the available data, ongoing research, and future directions in TFR in CML.

## 2. TFR Eligibility

There are many benefits of achieving TFR, making it the target of all current CML therapies. However, treatment withdrawal comes with risk of disease recurrence, and it is critical to first identify the patient populations most likely to retain sustained TFR prior to withdrawing TKI therapy and monitor them closely after the withdrawal of treatment. The positive sentiment towards striving for TFR is confirmed by recent CML patient survey data, which reveal that when asked about trialing TFR, a majority (61%) of patients are either willing or unsure about pursuing TFR, while 39% want to forego TFR and continue long-term with TKIs [12] Amongst patients who have undergone TFR, the most frequently cited concern (57%) is CML recurrence [13].

Finding specific eligibility criteria to delineate which patients should be offered TFR is essential. To date, there are two society wide recommendations on TFR eligibility. The most recent 2023 National Comprehensive Cancer Network (NCCN) guidelines recommend the following as patient criteria for discontinuation: an age >18 years, chronic-phase CML (CP-CML) with no prior history of accelerated-phase CML (AP-CML) or blast-phase CML (BP-CML), being on approved TKI therapy for at least 3 years with prior evidence of a quantifiable *BCR-ABL1* transcript and MR4 (*BCR-ABL* PCR ≤ 0.01% IS) for ≥2 years with reliable access to qPCR testing with a sensitivity of detection of at least MR4.5 (*BCR-ABL* PCR ≤ 0.0032% IS) that provides results within 2 weeks [14]. The 2020 European LeukemiaNet (ELN) guidelines overlap the NCCN guidelines but with more stringent recommendations. Notably, the ELN separates into minimally required criteria and optimal criteria prior to TKI cessation. Minimally required criteria include the following: the patient must be on first-line therapy (or second-line therapy if intolerance was only reason for changing TKI), typical e13a2 or e14a2 *BCR-ABL1* transcripts, a duration of TKI therapy >5 years (>4 years for second generation (2GTKI)), a stable molecular response (MR4 or better) for >2 years and no prior treatment failure. Optimal criteria for TKI cessation include the following: a duration of TKI therapy >5 years and a duration of deep molecular response (DMR) >3 years if MR4 or >2 years if MR4.5 [15].

These guidelines were formed from several studies, each conducted to find the optimal criteria to predict a successful TFR. The first prospective study designed to assess TFR was the Stop Imatinib Trial (STIM1), which evaluated 100 patients who had achieved undetectable (>4.5-log sensitivity) *BCR-ABL1* levels for >2 years [16]. Notably, inclusion criteria for most of the initial studies including STIM1, KID, TWISTER and ASTIM required undetectable *BCR-ABL1* levels for at least 2 years prior to TKI discontinuation, while more recent studies have trended towards more lenient inclusion criteria, with several only requiring sustained MR4 for 12 months prior to TKI discontinuation [16,17,18,19]. Included in the latter group is the EURO-SKI trial, the largest TKI cessation study to date, which provided helpful data to evaluate risk factors for TFR, as well as identify the risk of both early and late relapses [20]. A complete list of TFR trials to date with respective inclusion criteria can be seen in Table 2 [14,15,16,17,18,19,20,21,22,23,24,25,26,27,28,29,30,31,32,33,34,35,36,37]. On average, the molecular relapse rate was comparable between more stringent and more lenient discontinuation criteria, typically ranging between 28 and 44%, as will be discussed further.

While exact numbers vary, it can be estimated that of a group of CML-CP all-comers, about 10% will experience primary TKI resistance, 10% will experience secondary TKI resistance, 30% will experience a plateaued TKI response and only 50% will experience a deep response. While most CML-CP patients are interested in pursuing TFR, unfortunately, many are not eligible. To date, when determining if a patient is eligible to trial TFR, it is recommended to adhere to current national guidelines in close discussion with the patient in a shared decision-making context. That said, the expanding literature on this topic raises hope that we will eventually strike the delicate balance between liberal eligibility criteria and safe outcomes that will optimize the number of patients attempting TFR.

## 3. Strategies to Increase TFR Eligibility

As discussed previously, strategies geared towards increasing TFR eligibility are a rapidly expanding area. For most patients, the largest barriers to becoming TFR eligible are both obtaining and sustaining a DMR (>4 or 4.5 log reduction in a *BCR-ABL* product on international scale) [38,39]. Costa et al. have written a thorough review discussing various methods that have been attempted to increase rates of DMR and, thus, TFR eligibility [40]. While many different strategies have been employed to do this, they all largely fall into three main categories: maximizing the potency of upfront therapy, maximizing the tolerance of upfront therapy, and targeting CML leukemic stem cells (LSCs).

The strategy behind maximizing the potency of upfront therapy is based on the theory that more potent therapy will drive increased numbers of patients into DMR at quicker rates. This will then increase the number of patients eligible for TFR, as well as decrease the time it takes to get them there, albeit at times at the cost of increased adverse effects (AEs). Several of the specific strategies that have been attempted include upfront combination therapy, early TKI switching/proactive TKI switching and an induction/maintenance strategy. Regarding combination therapy, two trials (PETALs and TIGER) attempted to combine interferon therapy with a TKI. Both trials managed to show increased rates of DMR with combination therapy relative to TKI monotherapy; however, neither study showed any improvement in rates of sustained TFR after treatment discontinuation [41,42]. The FASCINATION trial showed impressive rates of MR4 at 12 months (38%) with the combination of asciminib and other TKIs, though it came at the cost of significant AEs (38% of patients had grade 3–4 AEs and 17% of patients discontinued therapy within first 12 months) [43]. With regard to early TKI switching, the DASCERN trial showed benefit in switching from imatinib to dasatinib after 3 months without response as patients who remained on imatinib had 12-month MMR rates of 13% compared to 29% in those who switched to dasatinib [44]. The SUSTRENIM trial attempted to see if early switching from upfront imatinib to nilotinib was equivalent to upfront nilotinib but found upfront nilotinib to be superior with higher rates of MR4.5 at 24 months [45]. The induction maintenance strategy is still in early stages of study without robust data. The IMPACT-I trial is a small single-arm trial that enrolled 10 patients to front line 2G-TKI until they achieved sustained MMR for 6 months prior to switching to imatinib. While the population size is small, the data are promising, with 89% of patients remaining in DMR on imatinib after >12 months with minimal AEs relative to 2G-TKIs [46].

The theory behind the second strategy of maximizing the tolerance of upfront therapy operates under the assumption that if a higher percentage of patients are able to tolerate the AEs associated with treatment, then an increased number of patients will stay on treatment longer and subsequently have a higher likelihood of qualifying for TFR. Multiple-dose de-escalation studies have been performed, attempting to find the minimal TKI dose required to sustain a durable DMR. Two notable studies include the DESTINY and DANTE trials. While both trials revealed that patients can sustain DMR on decreased TKI doses, it should be noted that in both trials, patients were started on standard-dose therapy prior to receiving decreased doses [21,47]. These results are helpful for patients requiring dose reduction in standard therapy due to AEs but do not provide insight into whether or not patients can receive reduced doses upfront. A more recent study attempted to answer this question by comparing upfront daily dasatinib doses of 100mg to 50mg and showed equivalent efficacy with fewer side effects in the lower dose [48]. While these results are promising, an ongoing study by Abraham et al. indicates that patients with high and intermediate risk disease determined via Sokal and ELTS scores may be less likely to respond to reduced-dose upfront therapy [49]. Further data are required to identify patients most likely to respond to reduced-dose upfront TKIs.

The last strategy attempting to increase TFR eligibility is to target LSCs. Unfortunately, it is a well-known fact that TKIs are not able to completely eradicate all LSCs even after a patient has achieved DMR [40]. By finding additional strategies aimed at removing these remnant populations of cells, the hope is that patients will be less likely to experience disease recurrence. This is still an area of much ongoing research, with only early data compiled to date. Several strategies are being looked at, including targeting LSC surface markers, apoptosis regulators and other actionable targets (JAK2, PPARγ, etc.); regulating bone marrow microenvironment; and epigenetic silencing. Virtually all these strategies involve employing a drug aimed at disrupting one or multiple pathways in addition to a TKI [40].

Given the wide variety of treatment options, it is important to acknowledge regimen-specific AEs and the impacts that they have on adherence and subsequent outcomes. With such effective treatment options available, it is not surprising that overall survival in CML is tightly correlated to regimen adherence. This was displayed by Kim et al. in their retrospective study that revealed that five-year overall survival varied between 70.2%, 85.2%, and 97.2% for low, moderate, and high TKI adherence, respectively [50]. As further treatment options become available, it will be important to find ways to maximize the tolerability of these regimens to optimize therapeutic potential.

Increasing the number of patients eligible for TFR is an important area of study with exciting data on the horizon. While it is possible that the most effective strategy may involve a combination of the three strategies discussed, it is also possible that with more data, treatment strategies will be selected based on patient risk factors and preference. In theory, high-risk patients may benefit from a shorter course of potent combination therapy, while low-risk patients may be able to obtain the same results with lower-dose therapy, thus minimizing AEs and increasing both tolerability and adherence.

## 4. TFR Efficacy

TFR efficacy is determined by the number of patients who can remain in sustained DMR without molecular relapse after TKI cessation. Many of the studies previously discussed utilized slightly different definitions of molecular relapse in their protocols. Table 2 summarizes both the definition of molecular relapse utilized and the rate of occurrence in each previously discussed study. A initial meta-analysis of 15 different TFR studies each using imatinib as first-line therapy revealed a 41% rate of molecular relapse at 6 months and a 51% overall rate of molecular relapse [51]. A more recent meta-analysis looked at 10 different TFR studies but included 5 studies that utilized 2G-TKIs as front-line therapy. This analysis revealed similar results of a 34% rate of molecular relapse at 6 months and a 41% overall rate of molecular relapse [52]. Given these findings, it is apparent that most molecular relapse occurs within the first 6 months, though later relapse can also occur. These data have influenced the current follow-up recommendations provided by both the NCCN and ELN to obtain monthly *BCR-ABL1* checks in the first 6 months, bimonthly checks in months 7–12 and checks every 3 months indefinitely thereafter [14,15].

While the data on molecular relapse in aggregate are promising, many studies have tried to investigate prognostic factors that may indicate both increased and decreased likelihood of sustained TFR amongst specific patient populations. Although each study provided an analysis of prognostic indicators based on their respective data, only a handful of these indicators are shared across multiple studies, as can be seen in Table 3. Between the two meta-analyses performed on TKI discontinuation, two prognostic indicators were found to have a consistent effect on sustained TFR: the duration of DMR and depth of molecular response before TKI discontinuation. More durable TFR was noted with >33 months of sustained DMR and patients who achieved MR4.5 [51,52] According to the 2019 meta-analysis, notable factors that did not seem to have a prognostic role were age, sex, Sokal score, the type of first-line TKI therapy, treatment history, TKI withdrawal syndrome and the total duration of TKI treatment before entering the TFR phase [52].

**Figure 1:**
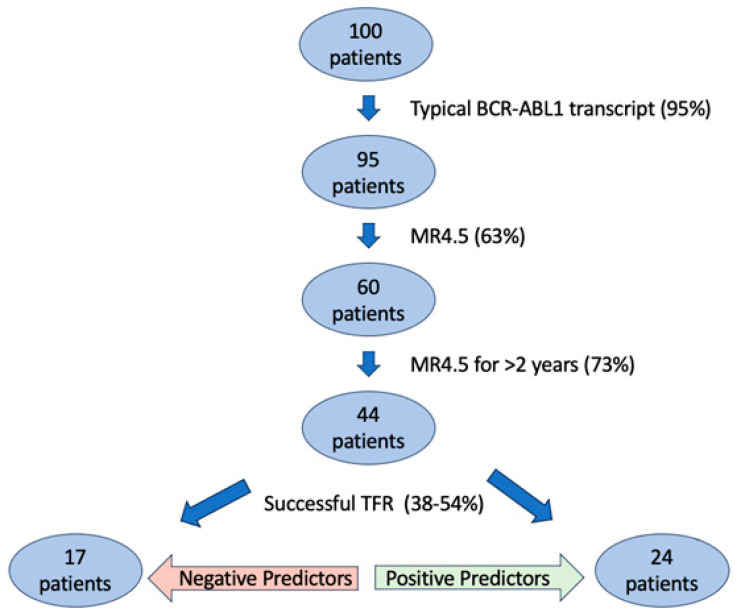
Likelihood of TFR success.

As more data on this subject are published, the ability to predict the likelihood of successful TFR in individual patients will increase, thus reducing possible risks that come with stopping TKIs in patients unlikely to respond to TFR. Presently, the actual percentage of patients estimated to undergo successful TFR is small. As an example, a hypothetical group of 100 patients with CP-CML hoping to attempt TFR based on optimal ELN guidelines can be utilized. Of this group of 100 patients, it is estimated that 95% of them (95 patients) will have a typical *BCR-ABL1* transcript [53]. Of those 95 patients, on average, only 63% (60 patients) will obtain a response of MR4.5 [54]. Of those 60 patients, it is likely that only 73% (44 patients) will sustain MR4.5 for 2 years and qualify to attempt TFR [54]. Drawing upon the various TFR studies, it can be estimated that overall rates of successful TFR range anywhere from 38 to 54% [16,31]. If applied to the hypothetical 44 patients who attempted TFR, that would leave between 17 and 24 of the original 100 CP-CML patients with successful TFR. While data to confirm the exact numbers are lacking, one could presume that a group of 100 patients with positive predictors of TFR success may yield closer to 24 successful attempts and a group without the same positive predictors may yield closer to 17. These data can be helpful to frame the likelihood of TFR success and can be seen in Figure 1.

## 5. TFR Failure

As outlined up to this point, the benefits of TFR are clear. That said, as with any medical intervention, it is important to acknowledge the risks associated with pursuing TFR as well. Most notably, it is important to understand what happens to patients who attempt TFR and fail. According to the meta-analyses performed by Campiotti et al. and Chen et al., no deaths were reported within the two-year follow-up of patients attempting TFR, only one patient amongst all the studies progressed to the blast phase and 98% of patients in molecular relapse regained MMR upon TKI re-initiation [51,52]. A third meta-analysis performed by Dulucq et al. reported that 90% regained DMR, which typically occurred within 6 months [55] These data are summarized by this study in Table 3. In all, TFR is widely accepted as safe given that even if a patient undergoes molecular relapse, they are unlikely to experience a progression of disease and have a very high likelihood of responding to TKI therapy again.

Given that the response to TKIs after initial TFR failure is so positive, it has led some to attempt second TFR. To date, the data on second TFR are not as robust. Only eight current studies have been conducted to look at the subject, with a total of 191 patients enrolled between the eight studies [31,56,57,58,59,60,61,62] Amongst those studies, the rate of a successful second TFR ranged from 20 to 41% at varying endpoints. Unfortunately, death rates, progression rates and rates of molecular recovery are not as thoroughly reported in all second TFR studies. However, according to the updated RE-STIM study, the above mentioned rates are comparable to the first attempt at TFR [59]. While the NCCN and ELN do not currently recommend second TFR attempts, further data on this intervention may increase the evidence that this is indeed a safe option for patients who qualify.

While not life threatening, additional detriments of TFR include the need for frequent *BCR-ABL1* checks indefinitely following TKI cessation and a TKI withdrawal syndrome associated with symptoms of musculoskeletal pain. A review of the adverse effects of TKI cessation written by Kota et al. suggests that these symptoms occur in 20–30% of patients who attempt TFR [63]. Symptoms are typically mild though can be severe enough to cause negative impacts on quality of life. In these instances, suggested management is NSAIDs, Tylenol and/or short course of low-dose oral glucocorticoids. In very severe cases, there have been instances of reinitiating TKIs after case-by-case discussions with the patient [63].

As previously discussed, laboratory checks after TKI cessation include monthly *BCR-ABL1* checks for the first 6 months, every other month from 7–12 months after cessation and every three months indefinitely after that. Though not the majority, a sizeable 31% of patients reported feeling anxiety regarding their follow-up laboratory checks [13]. Furthermore, the cost of these frequent checks should be noted. On average, each PCR costs about USD 150, which adds up to USD 1350 during the first year of TFR and USD 600 annually after that [64]. While this is not an insignificant amount of money, it should be noted it is still cheaper than the cost of lifelong TKI therapy at current prices, which can cost upwards of USD 150,000 annually [65].

## 6. Conclusions

In summary, TFR is an area of rapidly expanding research due to the promise it holds for allowing patients to safely remain off therapy long-term. Presently, the process by which TFR is initiated and discontinued is still in its early stages, and further data are needed for it to become fully optimized. Firstly, much benefit could be derived from increased clarity on TFR eligibility criteria. To date, there are disparate recommendations on TFR eligibility in the major clinical practice guidelines and an ever-growing body of literature with data that are not updated into formal guidelines in real time. The lack of uniform recommendations leads to concerns that individual practitioners may uptake differing interpretations of TFR eligibility, which could result in premature TKI cessation, thus minimizing the chances of successful outcomes. Secondly, more robust data to correctly stratify patients based on their likelihood of successfully completing TFR would be beneficial. By only attempting TFR in patients with a high likelihood of success, the rate of overall TFR success would increase, helping to alleviate patient anxiety, as well as prevent patients who are likely to relapse from stopping TKIs. Ultimately, many patients are not only hopeful to no longer require lifelong TKI therapy but also concerned about adverse events related to discontinuing their therapy. While much progress has been made, ongoing efforts to obtain data that further optimize the process of TFR will be of notable benefit to patients in the future.

## Figures and Tables

**Table 1:** Side effects and cost of tyrosine kinase inhibitors.

Drug Name	Notable AEs	Dose	Average Wholesale Price (USD, 2022)
Imatinib	Myalgias, arthralgias, skin rash, N/V/D, edema, renal insufficiency, pancreatitis	400 mg qD	USD 564–142,000
Dasatinib	Pleural/pericardial effusion, PHTN, QT prolongation, bleeding	100 mg qD	USD 228,000
		50 mg qD	USD 127,000
		20 mg qD	USD 63,000
Nilotinib	MI, peripheral arterial occlusive disease, diabetes, QT prolongation, pancreatitis, rash	300 mg BID	USD 240,000
		150–200 mg BID	USD 120,000
Ponatinib	HTN, HF, MI, stroke, peripheral arterial occlusive disease, arrhythmias, liver failure	15 or 30 or 45 mg qD	USD 271,000
Bosutinib	N/V/D, pancreatitis, increased ALT, renal insufficiency, rash	400 mg qD	USD 250,000
Asciminib	Arthralgias, dyspnea, dizziness, pancreatitis	40 mg BID	USD 258,000
		200 mg BID	USD 1,289,000

AEs: adverse effects; N/V/D: nausea/vomiting/diarrhea; qD: daily; bid: twice a day; MI: myocardial infarction; HTN: hypertension; HF: heart failure; PHTN: pulmonary hypertension; ALT: alanine transaminase.

**Table 2:** Inclusion criteria for TFR trials.

Trial	Treatment	Inclusion Criteria	Molecular Relapse Definition
STIM1 (*n* = 100)	Imatinib de novo (*n* = 50) or after IFN (*n* = 50)	UMRD for ≥2 years	Loss of UMRD
KID (*n* = 90)	Imatinib de novo (*n* = 82) or after IFN (*n* = 8)	UMRD for ≥2 years	Loss of MMR
TWISTER (*n* = 40)	Imatinib de novo (*n* = 19) or after IFN (*n* = 21)	UMRD for ≥2 years	Loss of UMRD
ASTIM (*n* = 80)	Imatinib de novo (*n* = 37) or after IFN (*n* = 42) or cytarabine (*n* = 1); combined therapy (*n* = 15)	UMRD for ≥2 years	Loss of UMRD
			Loss of MMR
ISAV (*n* = 108)	Imatinib de novo (*n* = 71) or after IFN (*n* = 36); 1 missing	UMRD for ≥1.5 years	Loss of MMR
EURO-SKI (*n* = 755)	Frontline: Imatinib (*n* = 710), dasatinib (*n* = 14), nilotinib (*n* = 33), unknown (*n* = 1); second line: imatinib (*n* = 7), nilotinib (*n* = 47), dasatinib (*n* = 62)	MR4.0 for ≥1 year	Loss of MMR
STOP 2G-TKI (*n* = 60)	Dasatinib (*n* = 30) or nilotinib (*n* = 30)	UMRD for ≥2 years	Loss of MMR
ENESTFreedom (*n* = 190)	Nilotinib	MR4.5 for ≥1 year	Loss of MMR
ENESTop (*n* = 126)	Nilotinib second line after imatinib intolerance (*n* = 51), imatinib resistance (*n* = 30) or preference for a physician (*n* = 45)	MR4.5 for ≥1 year	Loss of MR4
DADI (*n* = 63)	Dasatinib therapy after imatinib for resistance (*n* = 13), intolerance (*n* = 36) or a patient’s request (*n* = 14)	MR4.0 for ≥1 year	Loss of MR4
DASfree (*n* = 84)	Dasatinib frontline or subsequent therapy, no IFN	MR4.5 for ≥1 year	Loss of MMR
DESTINY (*n* = 174)	Imatinib (*n* = 148), nilotinib (*n* = 16) or dasatinib (*n* = 10)	MR4.0 for ≥1 year	Loss of MMR
		MMR for ≥1 year	Loss of MMR
LAST (*n* = 172)	Imatinib (*n* = 102), dasatinib (*n* = 27) nilotinib (*n* = 39) or bosutinib (*n* = 4)	MR4.0 for ≥2 years	Loss of MMR
DOMEST (*n* = 99)	Imatinib de novo (*n* = 83) or after IFN (*n* = 16)	MR4.0 for ≥2 years	Loss of MR4
D-STOP (*n* = 54)	Dasatinib frontline (*n* =19) or second line: imatinib (*n* = 34) or unknown (*n* = 1)	MR4.0 for ≥2 years	Loss of MR4
STAT2 (*n* = 78)	Nilotinib second line after imatinib (*n* = 73) or other treatment (*n* = 5); after IFN (*n* = 12)	MR4.5 for ≥2 years	Loss of MR4.5
STIM2 (*n* = 218)	Imatinib	UMRD for ≥2 years	Loss of UMRD
JALSG-STIM213 (*n*= 68)	Imatinib de novo (*n* = 55) or after IFN (*n* = 13)	MR4.0 for ≥2 years	Loss of MMR
First-line DADI (*n*= 58)	Dasatinib	MR4.0 for ≥1 year	Loss of MR4

IFN: interferon; UMRD: undetectable molecular residual disease; MMR: major molecular response; MR: molecular response.

**Table 3:** Outcomes of TFR trials.

Trial	Predictors of Response	Patients in TFR (%)	CML Related Death Rates (%)	Progression Rates (%)	MMR Recovery Rates (%)
STIM1 (*n* = 100)	Low-risk Sokal, longer treatment duration	38 at 60 months	0%	0%	96%
KID (*n* = 90)	Withdrawal syndrome, negative dPCR, longer treatment duration	69 at 24 months	NR	NR	100%
TWISTER (*n* = 40)	Low-risk Sokal, longer duration of IFN treatment prior to TKI	45 at 60 months	0%	0%	100%
ASTIM (*n* = 80)	Previous IFN treatment	44 at 36 months (loss of UMRD)	0%	1%	100%
		61 at 36 months (loss of MMR)			
ISAV (*n* = 108)	Older age, undetectable dPCR at time of TKI discontinuation	48 at 36 months	NR	0%	100%
EURO-SKI (*n* = 755)	Longer treatment duration, longer MR4 prior to TKI discontinuation	49 at 24 months	0%	0%	86%
STOP 2G-TKI (*n* = 60)	Prior intolerance or resistance to TKIs	54 at 48 months	0%	0%	100%
ENESTFreedom (*n* = 190)	Low-risk Sokal, longer nilotinib exposure, longer MR4.5	49 at 96 weeks	0%	0%	99%
ENESTop (*n* = 126)	Longer MR4.5 duration prior to TKI discontinuation	53 at 96 weeks	0%	0%	98%
DADI (*n* = 63)	Imatinib resistance, NK-cell counts, γδ+ T-cell count, CD4+ regulatory T-cell count	44 at 36 months	NR	0%	100%
DASfree (*n* = 84)	Older age, first-line therapy, longer treatment duration	46 at 24 months	0%	0%	96%
DESTINY (*n* = 174)	Longer treatment duration, MR4 group	72 at 36 months (MR 4.0 > 1 year)	0%	0%	100%
		36 at 36 months (MMR > 1 year)			
LAST (*n* = 172)	Undetectable RQ-PCR 3 months after TKI discontinuation, undetectable digital PCR at time of TKI discontinuation	61 at 42 months	0%	NR	96%
DOMEST (*n* = 99)	Longer duration of imatinib therapy, longer time from diagnosis to imatinib discontinuation, low-risk Sokal score	64 at 24 months	NR	NR	96%
D-STOP (*n* = 54)	None	63 at 12 months	NR	0%	100%
STAT2 (*n* = 78)	MRD negative prior to TFR	63 at 36 months	0%	0%	100%
STIM2 (*n* = 218)	NR	50 at 24 months	NR	NR	100%
JALSG-STIM213 (*n* = 68)	UMRD before TFR	65 at 36 months	NR	0%	100%
First-line DADI (*n* = 58)	Lower CD4-cell count prior to dasatinib discontinuation	55 at 12 months	NR	NR	100%

TFR: treatment-free remission; MMR: major molecular response; IFN: interferon; MR: molecular response; UMRD: undetectable minimal residual disease; dPCR: digital polymerase chain reaction; NR: not recorded.
